# The voice of the profession: how the ethical demand is professionally refracted in the work of general practitioners

**DOI:** 10.1186/s12910-023-00958-1

**Published:** 2023-09-26

**Authors:** Linus Johnsson, Anna T. Höglund, Lena Nordgren

**Affiliations:** 1 Centre for Research Ethics & Bioethics (CRB), Uppsala University, BMC, Box 564, 751 22 Uppsala, Sweden; 2 Department of Public Health and Caring Sciences, Uppsala University, BMC, Box 564, 751 22 Uppsala, Sweden; 3 Centre for Clinical Research Sörmland, Uppsala University, Kungsgatan 41, 631 88 Eskilstuna, Sweden

**Keywords:** General practitioners, General practice, Physician–patient relations, Ethics, Medical, Grounded theory, Sweden

## Abstract

**Background:**

Among the myriad voices advocating diverging ideas of what general practice ought to be, none seem to adequately capture its ethical core. There is a paucity of attempts to integrate moral theory with empirical accounts of the embodied moral knowledge of GPs in order to inform a general normative theory of good general practice. In this article, we present an empirically grounded model of the professional morality of GPs, and discuss its implications in relation to ethical theories to see whether it might be sustainable as a general practice ethic.

**Methods:**

We observed and interviewed sixteen GPs and GP residents working in health care centres in four Swedish regions between 2015–2017. In keeping with Straussian Grounded Theory, sampling was initially purposeful and later theoretically guided, and data generation, analysis and theoretical integration proceeded in parallel. The focal concept of this article was refined through multidimensional property supplementation.

**Results:**

The voice of the profession is one of four concepts in our emerging theory that attempt to capture various motives that affect GPs’ everyday moral decisionmaking. It reflects how GPs appreciate the situation by passing three professional–moral judgments: Shall I see what is before me, or take a bird’s-eye view? Shall I intervene, or stay my hand? And do I need to speak up, or should I rather shut up? By thus framing the problem, the GP narrows down the range of considerations, allowing them to focus on its morally most pertinent aspects. This process is best understood as a way of heeding Løgstrup’s ethical demand. Refracted through the lens of the GP’s professional understanding of life, the ethical demand gives rise to specific moral imperatives that may stand in opposition to the express wishes of the other, social norms, or the GP’s self-interest.

**Conclusions:**

The voice of the profession makes sense of how GPs frame problematic situations in moral terms. It is coherent enough to be sustainable as a general practice ethic, and might be helpful in explaining why ethical decisions that GPs intuitively understand as justified, but for which social support is lacking, can nevertheless be legitimate.

**Supplementary Information:**

The online version contains supplementary material available at 10.1186/s12910-023-00958-1.

## Background

Does general practice have an *ethical core* that remains stable while external moral demands and circumstances change? Given the myriad voices advocating diverging ideas of what general practice ought to be, can some “least common denominator” be distinguished that identifies a set of moral imperatives that GPs universally recognise as constitutive of their professional ethics, regardless of personal preferences, situational demands, and systemic constraints? 

It is not self-evident that general practice can find its ethical core in any of the traditions in which it is currently enmeshed. *Evidence based medicine (EBM)* has, despite its reassurances that “individual clinical expertise” should decide whether evidence applies to the patient [1], inspired little more than “guarded optimism” among GPs [2], and even GPs that regard EBM useful do not think that it exhausts the meaning of good clinical practice [3]. EBM has been regarded as increasingly “a science of marginal gains” that emphasises risk over disease [4], encouraging overtreatment [5] and harming both the sick and well [6]. Less redeemable features of EBM may be its attempts to provide scientific answers to some essentially non-scientific questions [7] and its doctor-centredness, which may be poorly compatible with patient-centredness [8].

Might the ethical core of general practice rather be found in the abstract moral principles of *bioethics*? Few physicians would today question the imperative to respect patients [9], and many moral problems are spontaneously conceptualised in terms of value conflicts, choice, and ethical dilemmas [10]. It is less clear that professionals agree with the emphasis that contemporary bioethics places on individualistic aspects of the doctor–patient relationship. As an example, the holistically inclined *patient-centred communication* taught to GPs differs significantly from the more intellectualistically tinged *shared decision making* [11]; and it is the latter that is implicitly underpinned by the bioethical norms of patient autonomy, preference satisfaction, and self-realisation [12]. 

*Virtue ethics* is a tradition that emphasises the development, through daily practice, of qualities conducive to wise choices [13], such as a “perceptual capacity” that allows them to judge properly [14]; it encourages “aligning reason and emotion so that the whole person acts rightly, as naturally as breathing” [15]. As many of its proponents can be found within the profession [13, 15, 16], virtue ethics might hold some claim to being intrinsic to general practice. Although there are some indications that GPs in certain settings make implicit use of it [10], it is thus far unclear how widely endorsed this tradition is within general practice.

Lastly, a rarely discussed yet influential ethical paradigm can be found in the phenomenological ethics of Løgstrup [17]. Its centrepiece is the *ethical demand*, a moral imperative based in the trust that we as humans show each other whenever we interact. Historically, phenomenological ethics has been embraced by care ethicists, perhaps because it harmonises with the “picture of the ideal nurse” who, unlike the doctor, “spontaneously shows caring behaviour” [18]; more recently, it has been found to be useful by physiotherapists [19]. It is at least possible that Løgstrup’s theory could prove relevant also to GPs, seeing as they interact daily with people who place a considerable part of their lives in their hands.

Although there are studies that describe substantively the morality of GPs in various situations, there have, to the best of our knowledge, been no attempts to integrate moral theory with empirical accounts of the embodied moral knowledge of GPs in order to inform a general normative theory of good general practice. In this article, we set out from our emerging theory of quality from the perspective of GPs [20, 21] to investigate the professional morality of GPs, understood as one of four main drivers of their ethical decision-making: the voice of the situation, which carries the problem as presented by the other, as well as their express wishes; the voice of the system, which relates the demands of people who are not currently present; the voice of the self, concerned with survival and thriving in one’s work environment; and the voice of the profession, which is the focus of the present article. We approach this matter descriptively by theorising those experiences and reflections of GPs that pertain to professional morality. To highlight the pertinence of such experiences to the moral discourse, we present a model that dresses them in moral terms rather than treating them as social or psychological facts. The model’s implications will be discussed in relation to previous research and ethical perspectives, especially principlism [22] and phenomenological ethics [17], to determine whether it might be, wholly or in part, sustainable as a general practice ethic.

## Methods

Our work followed a Straussian grounded theory approach [23] where data generation, analysis, and theory development took place in parallel, and constant comparisons were consistently used for discovering similarities and differences between exemplars. We used hypotheses and questions that arose during analysis to inform later iterations of data generation. Our metaphysical assumptions—that social concepts are real enough to be investigated [24] and that the purpose of inquiry is to provide justification for beliefs [25]—mark our stance as pragmatist.

### Population and participants

The study population consisted of general practitioners and GP residents working in Swedish health care centres. GP residents were included because they could be expected to share the commitment and ethos of their peers, while their relative lack of experience might provide interesting counterpoints.

Recruitment took place in 2015–2017. Potential informants were identified through personal acquaintance, at conferences, and through informal networks. We initially sought to include both men and women of different ages and levels of experience; later, theoretical sampling guided us toward contexts that might provide diverging data. All in all, we included eleven GPs and five GP residents working in eleven health care centres of varying sizes (from around 1,500 to 30,000 listed patients) in four counties. Both GPs and GP residents are henceforth referred to as “GPs” for readability.

### Sampling and data generation

Data were generated through observations and interviews. Each GP was observed during one half to one full working day. We thereafter conducted an unstructured interview in which we sought their reflections on present and past encounters, particularly with regard to their experience of quality in their work (see Supplement). Field notes were made during observations and expanded upon immediately after the interview. Interviews lasted 30–60 min, and were audiotaped and transcribed verbatim.

Transcripts and field notes were split and merged into discrete *events*, each of which detailed a central interaction and varying degrees of contextual information. In accordance with the tenets of symbolic interactionism [26], we paid attention to the meanings that the GPs ascribed to their actions. For the purposes of the present study, those meanings regarded the *abstract objects* that constituted their professional morality, no doubt formed through countless previous interactions of which we could catch only glimpses. We sampled 471 events before judging that theoretical saturation had been reached.

### Analysis and theoretical integration

One of the concepts that emerged from the data was the *voice of the profession*, a theoretical construct that helps make sense of judgments and intentions that are purely professional, as opposed to institutionally or socially imbued. Understanding this concept implies seeing clearly what kind of experiences it captures, but also what part it plays in the theory. Because the concept cannot be reliably connected to its exemplars without first understanding its function, which in turn is impossible without at least a preliminary definition of it, the activities of analysis and theoretical integration have necessarily run in parallel.

Analysis began with open coding. After the first few interviews and observations, we began forming preliminary categories and arranging them into a process, revising it as we gained new insights. The core category, which considers the different moral demands that inform the GP’s selection of a practical principle of action, was eventually described in an article [20].

By coding selectively around the highest-level concepts of the theory and simultaneously paying attention to the process, we eventually gained a deeper understanding of the circumstances that affect the moral actions of GPs. This led up to a second article in which we described the experience of stress—captured by the *voice of the self*—in general practice work, as well as the hypothesis that stress might guide the GP away from morally responsive action [21]. Although the *voice of the profession* is grounded in the same data as the previously mentioned concepts, we were able to elaborate on it only after coding selectively for it. While we did, a set of criteria emerged that could reliably identify exemplars. First, such exemplars take the form of moral imperatives, that is, things that ought or ought not be done. Second, they refer to something “larger than self” that transcends specific interactions. Third, they are “close to self” in the sense that they can be psychologically integrated regardless of what is socially expedient. Lastly, they indicate a potential conflict between the ideal and the practical, imlying that properly identifying values at stake but choosing not to act on them is a distinct possibility.

Variation among exemplars of the *voice of the profession* was accounted for through multidimensional property supplementation [27], a method that involves working back and forth between data and abstractions in order to discover a parsimonious set of orthogonal properties. Ideally, such a set should account for all *practically significant variability* and yield, when arranged geometrically, a comprehensive and comprehensible multidimensional typology. The model that we propose is consequently comprised of mutually exclusive subspaces that make those distinctions that are of interest while together accounting for all possible variation.

### Openness, sensitivity, and quality

The observations and interviews were carried out by LJ, himself a GP as well as bioethicist. He strived to maintain an insider perspective, empathising with the informants and co-authoring their stories in their voice. His pre-understanding was continuously made explicit in thousands of exploratory, methodological and theoretical memos. LN, with a background in nursing and considerable experience of conducting qualitative research, transcribed the interviews and participated in coding. Maintaining her distance to the interpretations, she was well positioned to question them and suggesting novel concepts. ATH is an ethicist with extensive experience in bioethical and qualitative methods as well as gender studies. She has focused on scrutinising the model and helping develop its relationship to extant descriptive and normative theory.

As this is the third paper in a series expounding different aspects of our emerging theory, our pre-understanding is infused with earlier findings and conclusions. This has had certain methodological implications for theoretical integration. While inductive reasoning has been paramount, in particular where the informants were forthcoming about their professional ideals, we have also made extensive use of retroduction [28], for instance when hypothesising professional ideals as explanations of what would otherwise be surprising behaviour. A GP might for instance appear to be unsatisfied, despite having given the patient what they came for while remaining well within the constraints imposed by the system; to make sense of their evaluation, one would need to hypothesise that they had sacrificed something else, such as an ideal.

The truth value of the *voice of the profession* depends largely on how it captures the experiences and behaviour of the target population. The model must *strike true,* naming professional norms in a way that is perceived by GPs as helpful while managing to introduce something novel and creative into the mix. Furthermore, the distinctions it makes must be theoretically clear, relevant, and helpful for discriminating between actual cases. For these reasons, member checking has been used to validate the model with regard to *fit* and *applicability* [23], two quality criteria that researchers might not be suited to evaluate themselves. Whether we have been successful in this regard must be ultimately judged by the initiated reader.

### Ethical considerations

Observations of doctor–patient encounters are potentially intrusive. This is more than a matter of confidentiality; the presence of an outsider might prove a detriment to the dynamic. For this reason as well as reasons of respect, all patients were given the option to refuse participating without offering any explanation. In addition, the informants (GPs) were asked to use their right to veto the researcher’s presence if deemed necessary to protect the patient. We were careful to not record any direct or indirect patient identifiers in field notes, and recorded health information only sparsely, mainly as an aid to recollection.

Although the interviews centred on professional experiences, some of these might also turn out to be deeply personal. Informants were therefore not pressured into telling us more than they were comfortable with. Because the informants might be identifiable from the recordings, the latter were handled confidentially and stored securely, and any identifiers were censored in the transcripts. Informants gave their oral and written informed consent beforehand.

## Results

**Fig. 1:**
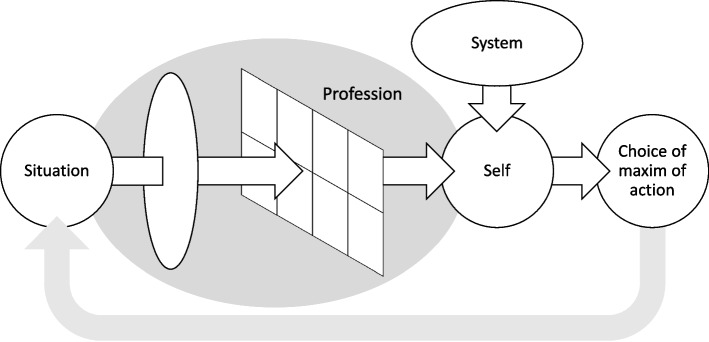
The ethical decision-making of the GP draws upon the demands of the voices of the *situation*, *profession*, *system*, and *self*. The *voice of the situation* carries the problem as it is presented by the other; the *voice of the profession*, the professionally embedded moral values and principles; *the voice of the system* relates the demands of people who are not currently present, including any institutional goals; whereas the *voice of the self* attempts to ensure survival and thriving in one’s work environment. The circularity of the process reflects the iterative nature of professional problem setting, intervention, and human interaction. The voice of the profession encapsulates a sub-process through which the GP identifies what moral values are currently at stake, thus framing the problem from the professional point of view

The *voice of the profession* is one of four concepts that attempt to capture the different types of motives that affect GPs’ everyday moral decisionmaking. What makes it peculiar is that it frames the concrete situation by appreciating it through a particular moral viewpoint. Through this sub-process, some moral values become emphasised at the expense of others (see Fig. 1).

### Three judgments that frame the moral problem

The GP’s professional understanding of the situation is formed by means of three basic moral judgments. Each judgment requires that the GP select one of two contradictory positions: Am I to meet present needs by *seeing what is before me*, or do I need to *take a bird’s-eye view* that includes the needs of people not currently present? Do I do so by *intervening* in the course of matters, or by *staying my hand* and letting events run their course? And do I need to *speak up* to influence people, or should I *shut up*, think and observe?

#### Seeing what is before me or taking a bird’s-eye view

When a GP stands before a patient in need, the imperative to *see what is before them* impels them to temporarily look away from the needs of other patients. Once the GP has decided on this perspective, it may not strike them as particularly problematic because those other needs are so much more abstract or hypothetical:

*I order those tests that I need … That doesn’t mean that I carry out investigations that are unnecessary. Because that is not good for the patient. … But I fail to see it as an economic problem. (Senior GP, male)*

In contrast, from *a bird’s-eye view*, the current issue becomes just one of many. Through this change in pespective, the GP may find that the overall pattern is what they should really be concerned about:

*I’m pretty strict when it comes to sticking to my schedule. I believe that consultations should be [max] thirty minutes … anything beyond that, and we should schedule a follow-up, out of respect for the next patient … (Junior GP, female)*

While both answers to the question recognise the need to balance values, the former envisions a much narrower scope within which such balancing is carried out.

#### Intervening or staying my hand

The imperative to *intervene* was ubiquitous in the observed encounters, which is unsurprising given that GPs are charged with curing illness and alleviating suffering. Interventions are also likely to be expected by the patient, which explains why some are not backed by medical motives, but rather serve to nurture trust:

*He didn’t really present any symptoms of diabetes, but … (sigh) … well, I thought of this as a way to create trust … I will need his trust for my model of explanation. (GP resident, male)*

Because many actions also carry destructive potential, knowing when to *stay one’s hand* is crucial. Although the one running the risk of being harmed is often the patient, this is not always so. One GP emphasised how GP residents must, when things are looking grim, learn to back down—and more importantly, do so with conviction, lest they lose hope in their abilities:

*It is dangerous to begin taking it personally, to think that ”I’m a bad doctor.” … Start thinking like that, and … you will easily become depressed about your work. (Senior GP, male)*

To embrace either answer is to take a stand on what one ought, metaphorically speaking, to do with one’s hands: to keep them busy or let them rest.

#### Speaking up or shutting up

The imperative to *speak up* tends to dominate whenever the GP stands in a position to influence (presumably for the better) how other people think. They may, for example, feel the need to recommend against surgery or other interventions in circumstances where the benefits would not justify the harms:

*When there is a history of substance abuse, they might be suffering from withdrawal and so on but once you’ve decided, you can’t change your mind … you need to be adamant … (Senior GP, male)*

*Shutting up*, in contrast, is necessary when the GP observes a process that should not be disturbed, for instance when a patient monologue is underway that reveals important information or serves some therapeutic purpose. Less obviously, the imperative to *shut up* is applicable to many situations where the GP has a clear mandate to decide on what needs to be done, which relieves them of the onus to argue their case:

*Back in the old days, people used to come in with myocardial infarctions and you name it … really ill people making a stopover. … So naturally, everything else had to be put on hold until you were done. (Senior GP, male)*

As either answer to the question demarcates a range of rhetorical strategies, this judgment is the one where GP ethics most clearly intersects with communication and consultation skills.

### The eight frames of GP ethics

By answering the three initial questions, the GP decides on a point of view that reveals what professionally endorsed values they believe are currently at stake. The binary nature of the questions allows a fairly simple model to be fashioned, the intention of which is to capture most, if not all, professional moral imperatives experienced by GPs. In this model, each of the 2^3^ = 8 possible combinations of answers corresponds to a frame. Considering each frame in the light of those events (encounters or parts of encounters) that it subsumes allows us to name and define them in a way that GPs should find evocative, while maintaining the robustness of the model as a whole.

The model of the *voice of the profession* is summarised in Table 1 and expanded upon in the following sections.

**Table 1:** By making three basic judgments, the GP frames the problem in professional terms. Their choice of frame reflects how their attention is called to different morally relevant aspects of the situation

	**See what is before me**		**Take a bird’s-eye view**	
	**Shut up**	**Speak up**	**Shut up**	**Speak up**
**Intervene**	**The patient is my first concern** I must cultivate those skills and relationships that I need to understand and address their problems	**I speak frankly and clearly** I must say what needs to be said to effect positive change, even when doing so feels awkward	**I apply myself with discretion** I must concern myself with and only with the most pressing issues, switching focus if and only if necessary	**I demand a better work environment** I must refuse to carry out tasks that do not require my skills, and demand to be assisted with those that I struggle with
**Stay my hand**	**I stand back and observe when there is time** I must not rush in or do anything in vain, but listen intently and weigh my words carefully	**I refuse to do harm** I must object to interventions that would cause disproportionate harm or incur unacceptable risks	**I enjoy being good enough** I must maintain a self-image that does not depend on the approval of others, celebrate my triumphs and refrain from destructive self-critique	**I uphold the integrity of my profession** I must protect general practice against undue expansion, rejecting responsibilities that would require me to quack

#### The patient is my first concern

When specific queues are lacking that would indicate a need to persuade anyone, refrain from harmful action, or consider any implications beyond the confines of the consultation room, the GPs is free to pursue their responsibility to make a difference for the patient before them in an active, focused, and verbally minimalistic manner.

Aware of the need to properly understand the nature of the patient’s ailment in order to address it, the GPs tended to work diligently to acquire the necessary tools for this purpose—mainly, their consultation skills and the relationship itself:

*Skilled surgeons operate, they spend time in the wound. … The list of patients is our wound, and that is what we should work on—learn to know our individuals and their diseases. And that is the preeminent condition of quality in primary care, to achieve doctor–patient continuity. (Senior GP, male)*

Several GPs attested to the importance of being able to postpone or abandon preconstructed (and possibly important) agendas in order to have time to address the patient’s concerns. Far from cutting corners, GPs considered this a worthwhile sacrifice as long as no danger was imminent:

*… she had done a lot of reading on the web and had lots of symptoms and ideas of what it could be, and somehow we ended up with a plan … Twenty minutes for a whole lot of things, so we didn’t do a physical examination but … she was calm when she left … (Junior GP, female)*

The above examples are united by specific answers to the three framing questions. First, the scope is narrow: The GP considers their main duty to be towards the person *before them*. Second, there is a need for active *intervention* in the sense that the GP must either provide a solution (for instance by prescribing) or work the problem into something that can be solved. Third, the GP *shuts up* in the sense that they do little in the way of argumentation, carrying out instead most of the work inside their own head. Since it prescribes duties that come naturally to GPs, this frame is in many ways the most relaxed.

#### I speak frankly and clearly

When GPs can do right by the patient only by making someone share their views, they consider themselves duty-bound to speak up rather than listen quietly. The duty holds even when the situation might become awkward.

Sometimes, the target of GP’s powers of persuasion was a third party, whom they attempted to influence for the benefit of the patient:

*… if I’ve made up my mind about putting a patient on sick leave for a month, then I’d better write a certificate that holds up to scrutiny. (Senior GP, male)*

More often than not however, the objective was to evoke a necessary change in the patient, be it with regard to their beliefs, emotions, or attitudes:

*… they went to the ER but were redirected here because they did not have a myocardial infarction … Some patients who get no help get more anxious. That chain will then be harder to break … That’s why I use a lot of pictures when explaining … (Senior GP, female)*

Like in the previous frame, the GP focuses narrowly on the patient *before them* and *intervenes* in their pursuit of good consequences. Here, however, the GP effects change by *speaking up*, bringing their reasoning into the sphere of interpersonal communication rather than keeping it private. Although this makes the current frame rather more demanding, many GPs seem to enjoy working within it.

#### I apply myself with discretion

Making the best possible use of one’s skills and capacities implies setting, and sometimes changing, one’s priorities. Less abstractly, GPs must sometimes switch focus from the matter before them onto other issues that are currently more important. At other times, they must reject requests the fulfilment of which would interfere with their current work.

Time being ever a limited resource, several GPs expressed the need to cope efficiently with lengthy patient agendas to avoid being sidetracked. While some spoke of “listening with half an ear” and reacting only to the most pressing matters, others preferred a more structured approach to agenda setting:

*You have to find out … Someone might be bringing a long list … “Well, this seems to be a lot … What do you find most important? … Can you name two things that you would like us to work on today?” You have to put it like that sometimes … (Senior GP, female)*

Even when being patient-centred, this informant as well as several others would not follow the patient blindly, but rather be prepared to switch tracks if there were any signs of significant risks to the patient’s health.

Unforeseen events outside of the consultation room required the GP to interrupt their consultations on a regular basis, and sometimes even to cut them short. Ensuring a safe work environment for their junior colleagues was universally a high priority:

*… I think it’s imperative that they feel comfortable asking … not erecting barriers that keep them outside … They are, after all, my future colleagues. (Senior GP, female)*

This frame differs from the first in that it prescribes looking away—at least temporarily—from the matter before one to take instead a *bird’s-eye view* of one’s work. Whatever the GP then decides to do—be it to drag themselves out of the present situation to attend to other issues, or to purposefully delay such a switch of focus—is quite obviously an active *intervention* with significant material consequences. Because the GP effects change through what they do rather than what they say, they generally *shut up* throughout the process, carrying out the ethical deliberation tacitly.

#### I demand a better work environment

When GPs become obstructed in their pursuit of worthy goals, they find themselves obligated to work around the obstacles in their path. Because the less worthy tasks have to be carried out before the GP can apply themselves to what truly makes a difference, the problem cannot be managed by prioritising; instead, they need to persuade others to assist them.

Requesting manual assistance with procedures seemed to incur some risk of creating friction in the workplace. Several GPs grumbled about having to spend valuable time cleaning or searching for equipment, but did little to change these states of matters. Even when lagging behind their schedule, few actually raised their voice:

*I oversee 75 patients in home health care. Now, some diagnosis must be removed … it’s an administrative thing. … I would be better off seeing a patient or something. (Junior GP, female)*

The GPs were also aware of—but did not always try to enforce—their right and duty to educate themselves, a crucial investment that would nevertheless, due to perpetual shortages of staff, risk being put on hold indeterminately:

*… the intention of management is, I suppose, that everyone should get time off and educate themselves, stay up to date, but for the past year it hasn’t … We’ve not received much continuing education becaused we’ve been understaffed … (Junior GP, female)*

The present frame involves a *bird’s-eye view* of different aspects of one’s work environment, and prescribes *intervening* to change it for the better by *speaking up* about unmet professional needs. Judging from the words and actions of the observed GPs, they have a hard time living up to their own ideals in this respect.

#### I stand back and observe when there is time

The ethical competence of GPs necessarily includes reflective awareness of their potential to inflict harm as well as doing good. The main consequence of this awareness is the realisation that sometimes, the best course of action is to intervene minimalistically, if at all.

Forgoing prescribing drugs when the patient’s condition appeared to be self-limiting was the most common (and almost trivial) form of minimalism. More interestingly, minimalism would sometimes be reflected not in what actions they authorised, but in what they communicated. For instance, a GP meeting with a patient expecting medically superfluous interventions would face a balancing act where they had to attribute at least some (and sometimes considerable) weight to the risk of disrupting the relationship. One GP regarded playing the long game as more important than seeking short-term victories, at least when the intervention in question was perceived as relatively harmless:

*… to some degree, you have to adapt and approach the patient in the way that they expect health care to be. … some people are used to … always having samples taken. … you might have to take some samples … next time, when I say, “I do not think that will be necessary,” then, “Ok.” (Senior GP, male)*

Some GPs spoke about taking care not to jump to conclusions, even during seemingly uncomplicated emergency appointments. By employing a “dormant vigilance,” they could remain mostly relaxed yet constantly on the lookout for signs of serious disease. Observing quietly could also be an effective strategy for grasping enigmas, as spontaneously offered bits of seemingly unrelated information might make sense of even the most mystifying symptoms, often in a flash of insight:

*… for the past couple of days, her shoulder had been drooping. “Have you been in an accident?” “No, I just noticed it in the mirror a few days ago.” … Completely inexplicable. … And then her husband says, “And there was this other thing, about her short-term memory.” … And then it all made sense … (Senior GP, female)*

Like the first frame, the present one is about what occurs *before one* as well as about *shutting up* and listening, here motivated by the insight that careless words might irreversibly disrupt the picture. Where it differs is in prescribing *staying one’s hand* in order to see how things are developing before intervening. By melding into the backdrop rather than taking centre stage, the GP becomes well situated to learn and to gain trust, at the cost of temporarily sustaining the tension or partly relinquishing control over the flow of events.

#### I refuse to do harm

Occasionally, GPs are asked to intervene in ways that they believe would incur a risk of harming the patient. In such situations, the expectations of others cannot be problematised with mere passive resistance; instead, the GP must be vocal in their opposition.

Straightforward examples of refusing to do harm concerned prescribing drugs with potentially severe side effects, or exaggerating loss of function in the sick-leave certificate:

*… I ramped up her work ratio a little. She seemed a bit reserved about that, perhaps not completely satisfied, but I think she can make it … You might falter somewhat and, like, drag out the sick leave full time for a bit too long. (Senior GP, male)*

Other forms of refusal required the GP to stand up to the system rather than the patient, as when mandatory interventions made little sense and might even be harmful:

*… we are supposed to ask everyone about suicidal ideations … Those are questions that need to be asked in the right context … You can hardly prevent all suicides in that manner. … I think there are many who would be put off or even insulted … (Senior GP, male)*

Other forms of refusing to do harm were interlinked with questions about competence. When lacking the knowledge necessary to safely handle the patient’s condition, for instance, many GPs would feel morally compelled to be frank about their shortcomings.

The professional imperatives in this frame are united by, first, undistracted attention to *what is before one*; and second, refusing to act, which amounts to *staying one’s hand* while *speaking up* to defend one’s decision to potentially critical observers.

#### I enjoy being good enough

Although doing well by the patient might be the main driver in GP ethics, this pursuit is tempered somewhat—especially among senior GPs—by the grim realisation that some conditions are incurable, some suffering difficult to alleviate, and some deficiencies of the system impossible to compensate for. Enjoying being good enough in spite of such limitations appears to be a prerequisite for a sustainable professional life.

Senior GPs commented on the tendency, seen mostly among younger doctors, of “listening for zebras” or, more generally, ordering extensive testing in order to be “safe,” where such excesses would sometimes in fact leave the patient worse off. A perhaps more interesting motive to help them to hold back was concern for their professional development:

*… you have to accept that … you don’t know everything and that you will make mistakes. If you … can’t make up your mind, if you become anxious … you can’t stay in this field. (Senior GP, male)*

On the flip side of accepting one’s limitations, the GPs appeared to allow themselves to celebrate—albeit between clenched teeth—near-failures that ended in triumph.

A stable professional role does not imply rigidity. One GP endorsed preparedness to revise their decisions in the face of new evidence—sometimes no more than a gut feeling—even though their imperfections might thus be exposed:

*Once I had made up my mind, I used to be rather unflexible … After a while, I realised that … it made me feel bad and it was dangerous. … I could make things much easier for myself by … doing that extra checkup, seeing the patient again … (Senior GP, male)*

The kind of focus on one’s own professional role that we see in this frame implies taking a *bird’s-eye view* and deciding to *shut up* and *stay their hand* instead of making futile (and potentially destructive) attempts at improving what is already good enough.

#### I uphold the integrity of my profession

The last frame is about the limits and boundaries of the professional role of GPs—in other words, about general practice itself. It captures the duty of the GP to protect their profession against demands that if met (at least systematically) would threaten to dilute it or otherwise deprive it of legitimacy.

The GPs regarded many demands as dubious, resulting from societal trends toward patient emancipation and medicalisation of everyday life. To them, upholding the integrity of their profession seemed to require, first, that they be perfectly clear about the limits of the patient’s rights, which would depend on their actual health care needs as well as justice considerations, and second, actually enforcing those limits. Ideally, they would do so with a soft touch:

*Usually, I try to say to the patient, “Begin by describing your problem and then we … will try to help you find the right way …” (Senior GP, male)*

Several GPs attested to a form of systematic disrespect from their hospital-based colleagues, who seemed to expect them to assist with housekeeping tasks such as ordering specific tests and examinations. Some even attempted to formulate defensive principles against the onslaught:

*… it’s reasonable that the one who wants an examination to be carried out also will be the one to order it, isn’t it … (Senior GP, male)*

In general, their hospital-based colleagues saw fit to maintain very strict boundaries around their own competence while expecting the GP to dabble.

In the face of critique from outside their practice, the GPs might still find some strength in their professional norms, particularly when the critique was unfair or irrelevant:

*… what we are assessed by are usually these simplistic parameters, like … how many patients with atrial fibrillation are on warfarin, for example … for individual patients, other things may be much more important. (Senior GP, male)*

The GP meets outside critique—be it overt or latent—by taking a *bird’s-eye view* of their professional practice and *speaking up* to defend their decision to *stay their hand* where others would expect them to act. Being one of the most combative ones, this frame entails significant strain on those that choose to meet the duties that it prescribes.

## Discussion

The GP is subject to a multitude of demands from several sources: the *voice of the situation*, which captures explicit demands voiced in the situation; the *voice of the system*, which lends support to some actions and discourages others; the *voice of the self*, which accounts for the stress that results from threats to the GP’s personal needs [21]; and the *voice of the profession*, which captures a professional moral view that does not depend directly on the situation or context.

Our findings indicate that the work of GPs is at a constant risk of becoming unmanageably complex, and that GPs may have a method for reducing this complexity. By selecting a “frame” that reveals a range of actions that fulfil the currently most pertinent moral values, GPs may hope to narrow down the range of possible ethical considerations. In a sense, such framing is an example of *problem setting* [29] by which the professional transforms a problematic situation into a workable problem.

### The voice of the profession in a wider context

By being sensitive to concrete particulars, the *voice of the profession* takes a wide-angle view of the morality of GPs, one where tenets assumed to be central to general practice occupy only a small portion. Take, for example, the undoubtedly crucial concept of *patient-centredness* [8]. One of its implications, that one should strive to know one’s patients as individuals [30], is clearly visible in *The patient is my first concern*; another one, which dictates that one should refrain from being doctor-centred, is highly relevant in *I stand back and observe when there is time.* Within the remaining six frames, however, the concept is rather silent.

The case is markedly different with *virtue ethics.* While the three other voices require little or no virtue on behalf of the GP, its relationship to the *voice of the profession* is intimate: perceptual capacity [14] is arguably a prerequisite for framing in the first place, whereas character virtues such as justice, courage, and truthfulness [31] seem all but indispensable in order to heed the call. Although virtue ethics may bring many insights regarding what ends should be valued and what means should be obtained, the ubiquity of the virtues also entails that they may not be of much use analytically in this article.

In what follows, we shall consider our findings in some detail in the light of two different moral theories: *principlism* [22] and *phenomenological ethics* [17].

#### Principlism

The four-principle model of Beauchamp & Childress [22], sometimes referred to as *principlism*, has become rather popular in medical ethics. Its principles—*respect for autonomy*, *non-maleficence, beneficence* and *justice*—are empirically as well as theoretically grounded and purport to capture most, if not all, relevant concerns that may arise in moral quandaries within the medical field.

How does the *voice of the profession* fit with principlism? On a semantical level, the four principles can certainly be recognised in our model. Beneficence underpins *seeing what is before me*, whereas *taking a bird’s-eye view* draws more upon its close cousin, utility, as well as justice. *Staying my hand* is generally motivated by non-maleficence. Things are trickier with autonomy: while we might construe *shutting up* while *seeing what is before me* as respect for autonomy in a negative sense, *speaking up* seems necessary to meet positive requirements such as adequately informing the patient.

When it comes to accounting for the *process*, however, there are a few discrepancies between how principlism envisions ethical deliberation and how such deliberation appears to play out in practice that indicate that principlism may not help us understand how GPs in fact handle everyday moral problems.

1. *Limited salience*. Within some frames, a single principle dominates to the exclusion of the others. For example, in *I speak frankly and clearly*, beneficence potentially infringes on autonomy; *I refuse to do harm* unsurprisingly lets non-maleficence outweigh both beneficence and autonomy; and *I apply myself with discretion* draws mainly upon procedural, comparative conceptions of distributive justice. Once the problem has been framed, questions of right and wrong action are therefore already all but settled. Due to its focus on moral decision-making, it is clear that principlism must be complemented with value negotiation and reflection skills in order to be useful in the arguably more important activity of moral problem setting.
2. *Limited output value.* In frames where several principles are salient, it is questionable whether they by themselves generate any useful advice. *The patient is my first concern*, for example, may be oriented towards beneficence, but also speaks from an awareness that careless words might cause harm; furthermore, its idea that sincere presence (rather than mere lack of interference) is required is a nod to autonomy in the positive sense. However, to the degree that the GP recognises that this is what is required of them, they hardly gain this insight solely by recounting the four principles. All moral rules require specification to be effective [22]; but in the case of GPs, the skills that they need for this purpose are external to the theory, as they consist in attending to the specifics of the situation in the light awarded by deep contextual knowledge.
3. *Limited comprehensiveness.* Some frames appear to speak of matters alien to principlism. In *I enjoy being good enough*, the master is heard gently admonishing the apprentice for being overly self-critical. Although greater utility for future patients may well follow, it is clearly the GP’s professional life, not that of the patient, that is centre stage here. Similarly, *I demand a better work environment* and *I uphold the integrity of my profession* go well beyond any implications of the four principles as they attend to specific, morally relevant circumstances of the GP’s work.

What are the implications of this lack of fit? Since the intention of principlism is normative, one might argue that the *voice of the profession*, to the degree that there is disagreement, lacks normative power. Locating ethical deliberation within the process of framing does not relieve the GP from the imperative to weigh moral principles against each other. It seems to follow that the empirical evidence presented in this article only indicates that this weighing must take place during framing rather than later in the process.

The assumption that the moral imperative must necessarily take on the guise of principlism may turn out to be unwarranted, however. Other authors have noted how a “quandary ethics” approach may fail to account for mundane yet pervasive forms of professional moral deliberation [32, 33]. The ethical training of GPs is mostly practical, consisting in them being constantly forced to respond to problematic situations by passing judgment on them, constructing from them workable problems. It should be safe to assume that a GP with the right set of motivations will become rather good at this. If a particular theoretical model of deliberation— be it principlism or something else entirely—does not rhyme with their process, forcing its adoption might not be very helpful. The onus is therefore on proponents of principlism to explain why its logic, although at odds with how GPs actually think, should be the preferred form of thinking.

After considering the relative merits of principlism, and conceding its usefulness in addressing moral quandaries, we believe that the reader should feel compelled to look elsewhere for a suitable theoretical counterpoint to everyday GP morality.

#### Phenomenological ethics

The *phenomenological ethics* of Løgstrup conceives of an *ethical demand* to take care of the life of the other such as it has been delivered up to one [17]. At first glance, this idea would seem to harmonise best with those frames of the *voice of the profession* that are explicitly concerned with a caring relationship, such as *The patient is my first concern*. Beneath the surface, however, there is a more profound agreement that might help us understand the overall intentionality of the *voice of the profession.*

First of all, our idea of conflicting voices is compatible with Løgstrup’s assertion that the ethical demand remains stable as social norms change, yet is sensitive to concrete relational and situational aspects. This does not imply that the ethical demand itself changes; rather, it is *refracted* through three lenses: the relationship, the situation and the self. We would here argue that the *voice of the profession* incorporates a special case of Løgstrup’s relational lens, refracting the way it does because of the peculiar character of the doctor–patient relationship. In this view, the act of framing is the GP’s professionally imbued way of determining how the other is best served.

Another compatibility follows from the idea of the ethical demand as *unnegotiable* yet *silent.* On this understanding, no imperative can be formulated ahead of the fact; instead, it is the responsibility of the trustee to discover, through their understanding of life, how they might best serve the other. Doing so might very well run counter to the express wishes of the other; “being nice” when the other would be best served by denying their request equates to pandering and is a sign of an insincere relationship. This line of thinking predicts a potential for conflict between the *voice of the profession* and the *voice of the situation.* That their demands often coincide belies the fact that heeding the command of the *voice of the profession* implies that the GP makes a proper judgment regarding the values at stake and then acts on that judgment, regardless of any conflicts that might be thus provoked.

Its merits notwithstanding, phenomenological ethics has been regarded as problematic because focusing on the needs of “the other” may cause one’s obligations to “the third” to fall out of the picture [18]. It is a fair question whether phenomenological ethics is able to say anything substantial about bird’s-eye matters such as priority setting and protecting the integrity of the profession. While a full inquiry on this topic is certainly out of this article’s scope, we shall suggest a way that a conception of justice might be incorporated.

Let us first agree that through his distinction between taking care of and pandering the other, Løgstrup also claims that denying explicit requests is sometimes what is morally required. While this is undoubtedly one of the preconditions for a reasonable conception of justice, it is not enough. To be precise, it does not explain how such denial—obviously motivated by concern for the *third*—could ever be conceived as also serving the *other*.

The problem, however, may be illusory. As long as the third remains a distant, abstract possibility rather than a person in their own right, we will tend to discount the possibility that the ethical demand could ever apply to them. Once the third is there in the flesh however, it becomes obvious that they are also an other who must be served. Now, to the GP who meets many patients every day, this is a concrete fact of life; to them, it rarely makes sense to understand the weight of one patient’s needs in isolation from their experience of previous patients or projections of future encounters. The third can thus be as deserving of being cared for as the other; any reservations that one might have on this matter derive from uncertainty regarding their needs, where in contrast those of the other are in plain sight. Although much theoretical work remains before this matter can be settled, envisioning obligations to a third person through Løgstrup’s phenomenological ethics no longer seems impossible.

One last comment must be made regarding the GP’s obligations to the profession itself. While some of the frames, in particular *I demand a better work environment*, formulate imperatives that harmonise with the interests of the *voice of the self*, they do not collapse. The distinctly professional take on these matters is that certain actions may be necessary if the GP is to be able to work sustainably. On this understanding, professional autonomy is not merely about one’s personal interest in being unrestrained, nor is professional integrity simply about feeling good about what one does. To the contrary, both are fundamental aspects of the doctor’s ability to respond to current and future patients.

### Strengths and limitations

A high-quality grounded theory should allow the reader to understand the predicaments faced by specific groups of people, and possibly to explain their actions relative to some phenomenon. The main strength of this study is the relevance of the *voice of the profession* in our emerging theory about GPs’ moral decision making. It is also possible that GPs will find the concept a useful tool when reflecting on their everyday moral decision making. They might, for instance, attempt to analyse their moral stance towards particularly troubling cases by reflecting on what frame they were working in, and whether some other frame might have been more fruitful.

Several methodological decisions have contributed to this study’s quality. Through constant comparisons, we have been able to discover and theorise similarities and differences between a large number of exemplars. Sampling has been purposeful—drawing from the relevant population—as well as theoretically guided, and the use of triangulation has reduced the risk of misinterpretation or misrepresentation. The use of multidimensional property supplementation [27] has helped make the focal concept comprehensive and robust. The respective pre-understandings of the three authors have been rather different, mitigating the risk of biased interpretations.

Due to the complexity of the focal concept, certain aspects that may be important to fully appreciate its function in the main process have been here treated superficially. We have not, for instance, expanded on the ample room for conflict between the *voice of the profession* and the other three voices. Although it is easy to imagine that the outward appearance of acting *idealistically* (in line with professional demands) or *pragmatically* (in opposition to them) will differ depending on where the battle lines are drawn, we have been unable to spell out all implications here.

While the ambitions of grounded theories with regard to normativity are usually rather limited, we have dared make the claim that the *voice of the profession* should also be understood as normative. This may be a cause of concern for some readers, especially perhaps those with deep roots in normative ethics. One reason not to dismiss our empirically derived theory as merely descriptive is that professional morality, to the degree that it has evolved through the engagement of many professionals within a shared practice, is likely to *work*. Another, related, reason is that it presumes no particular social context or set of social norms. In fact, given enough abstraction, we believe that it may transcend time and culture.

## Conclusions

The *voice of the profession* is a concept that captures several essential features of GPs’ moral decision making. By framing an initially problematic situation in moral terms, the GP narrows down the range of ethical considerations, enabling them to focus on its morally most pertinent aspects. The moral imperatives spoken by the *voice of the profession* sometimes stand in opposition to the express wishes of the other, social norms, or the GP’s self-interest.

In its strictly deductive approach and focus on ”moral quandaries,” *principlism* looks away from the idiosyncracies of the situation. Its gaze can thus become blind to the tensions between the respective wills of the patient, institution, and profession. Moreover, principlism may misrepresent the actual process of moral decision-making, where a significant portion of the deliberation consists in making judgments about what the situation requires of the GP in terms of seeing, acting, and speaking.

Reading our findings through Løgstrup’s *phenomenological ethics*, in contrast, allows us to see the act of framing as a form of refraction of a fundamental ethical demand through a professional–relational lens, whereby the GP discovers what is more specifically demanded of them. It also becomes clear why, in spite of the GP’s professional understanding of what is morally at stake, demands from other sources are not eliminated, but continue to exert pressure on the GP’s self. In sum, the *voice of the profession* is coherent enough to be sustainable as a general practice ethic, and can be helpful in explaining why certain ethical decisions that GPs intuitively understand as justified, but for which social support is lacking, can nevertheless be legitimate.

### Supplementary Information

**Additional file 1.**

## Data Availability

The data that support the findings of this study are available on reasonable request from the corresponding author. The data contain information that could compromise research participant privacy, and making the data publicly available would violate the terms of their consent.

## Abbreviations

EBM: Evidence Based Medicine
GP: General Practitioner
