# Supplementary material for: The voice of the profession: how the ethical demand is professionally refracted in the work of general practitioners
Source: BMC Med Ethics. 2023 Sep 26;24:75. doi: 10.1186/s12910-023-00958-1 (PMC10523728; doi:10.1186/s12910-023-00958-1)
Supplement: Supplementary file 1 — Additional file 1. [file 12910_2023_958_MOESM1_ESM.docx]

Quality from a general practitioner’s viewpoint

Interview guide (translated from Swedish)

The following areas of inquiry indicate the boundary for the kind of questions that will potentially be asked during the interviews. The areas follow directly from the research questions.

Covering all areas during every interview is neither necessary nor desirable. The hypotheses that emerge, and the gaps in the theory that become apparent, during the course of the project will decide what questions are currently pertinent.

# What concrete experiences does the participant have of quality or lack of quality in their work?

This area includes mainly ”What”-questions. A natural point of departure would be those events that the participant and the researcher have experienced together during the day, but other events can also be discussed. Of particular interest are experiences with a strong positive or negative valence.

**Examples:** ”Was there any occasion during the day when you felt particularly satisfied/unsatisfied with your work? Can you tell me more about that? What happened then? What did you feel at that point? Can you recall other situations where you have experienced something similar?”

# In what way is quality challenged in daily work, and how does the participant resolve these conflicts?

Here, ”How”-questions dominate. The participant is encouraged to retell their experiences in terms of causes, decisions, and consequences. Special attention is paid to interactions with other actors.

**Examples:** ”When did quality come to a head in the situation that you have told me about? What was it that led up to the conflict/problem? Was there anything in in particular the other’s behaviour that made you react? What were the consequences of your actions?”

# How does the participant understand the concept of quality?

These are ”Why”-questions because they concern one possible cause of the participant’s conduct and emotional responses in various situations. We are hence looking for an implicit conception of quality. This part of the interview is particularly difficult due to the risk of sliding into theoretical speculation.

**Examples:** ”In what way is what you have told me about an example of good/poor quality? What was it that was crucial for quality? Can you envision something that could have dramatically altered this experience? Can you provide examples of situations where similar actions on your part rather led to poor/good quality?”
